# Supplementary material for: Current Indications and Future Landscape of Bispecific Antibodies for the Treatment of Lung Cancer
Source: Int J Mol Sci. 2023 Jun 7;24(12):9855. doi: 10.3390/ijms24129855 (PMC10298459; doi:10.3390/ijms24129855)
Supplement: Supplementary file 1 [file ijms-24-09855-s001.zip › ijms-2259307-supplementary.pdf]

3F8: Humanized anti-GD2/anti-GD3 bispecific antibody. GD2 is a disialoganglioside expressed in several tumors including neuroblastoma and melanoma, with low expression in healthy tissues.

68Ga-THP-APN09: ^{68}Ga labelled nanobody.

89Zr-AMG211: ^{89}Zr labelled BiTE directed against carcinomembryogenic antigen (CEA) and CD3.

ABL503: Bispecific antibody that combines PD-L1 inhibition and 4-1BB agonistic activity. 4-1BB is expressed on the surface of leucocytes and induces T cell proliferation, B cell antibody secretion and dendritic cell maturation upon its interaction with 4-1BBL.

AFM24: Tetravalent bispecific recombinant antibody targeting EGFR and CD16A. CD16A is expressed on the Surface of mast cells, macrophages and natural killers.

AGEN1223: bispecific antibody targeting two antigens associated with Tregs still undisclosed, causing Treg depletion.

AK104: Humanized IgG1 bispecific antibody targeting the inhibitory immune checkpoints PD-1 and CTLA-4.

AK112: Humanized IgG1 bispecific antibody targeting PD-1 and VEGF.

AK129: Bispecific antibody targeting PD-1 and lymphocyte-activation gene 3 (LAG-3). LAG-3 is an inhibitory immune checkpoint expressed on activated T cells, NK cells, B cells and dendritic cells, which modulates T cell activities.

AMG 757: Half-life extended BiTE targeting delta-like ligand 3 (DLL3) and CD3. DLL3 is an inhibitory Notch ligand overexpressed in neuroendocrine tumors.

Amivantamab: Human IgG1 bispecific antibody targeting EGFR and MET.

ATOR-1015: Human IgG1 bispecific antibody that combines inhibition of CLTA4 and OX40 agonistic activity. OX40, when binding to OX40L, activates T cell and promotes expansion of effector and memory populations.

AZD2936 (**Rilvegostomig**): Monovalent humanized IgG1 bispecific antibody targeting PD1 and T cell immunoglobulin and ITIM domain (TIGIT). TIGIT is an inhibitory receptor expressed in lymphocytes that interacts with CD155 on antigen-presenting cells, downregulating T cells and NK cells activities.

AZD7789: Bispecific antibody simultaneously inhibiting PD-1 and T cell immunoglobulin and mucin-domain containing protein 3 (TIM-3). TIM-3 is an immunoregulatory protein expressed on T-cells, Tregs and other immune cells.

B1962: Bispecific antibody targeting PD-L1 and VEGF.

BAT7104: Bispecific IgG-like antibody targeting PD-L1 and CD47. CD47 is a tumor-associated antigen usually found on the surface of healthy cells but overexpressed on cancer cells, which inhibits macrophage activation.

bi-4SCAR GD2/CD56 T cells: 4th generation bispecific CAR targeting GD2 and CD56. GD2 is a disialoganglioside expressed in several tumors including neuroblastoma and melanoma, with low expression in healthy tissues. CD56 (NCAM-1) is highly expressed in cancer with neuroendocrine differentiation.

bi-4SCAR GD2/CD70 T cells: 4th generation bispecific CAR targeting GF2 and CD70. GD2 is a disialoganglioside expressed in several tumors including neuroblastoma and melanoma, with low expression in healthy tissues. CD70 is a ligand for the coestimulatory receptor CD27, it is expressed on the surface of some cancer cells and is associated with immune evasion.

bi-4SCAR GD2/PSMA T cells: 4th generation bispecific CAR targeting GF2 and prostate-specific membrane antigen (PSMA). GD2 is a disialoganglioside expressed in several tumors including neuroblastoma and melanoma, with low expression in healthy tissues. PSMA is expressed in prostatic tissue and overexpressed in prostate cancer, as well as in other tumor types.

bi-4SCAR PSMA/CD70: 4th generation bispecific CAR targeting PSMA and CD70. CD70 is a ligand for the coestimulatory receptor CD27, it is expressed on the surface of some cancer cells and is associated with immune evasion. PSMA is expressed in prostatic tissue and overexpressed in prostate cancer, as well as in other tumor types.

BL501: Bispecific antibody targeting PD-L1 and LAG-3. LAG-3 is an inhibitory immune checkpoint expressed on activated T cells, NK cells, B cells and dendritic cells, which modulates T cell activities.

BNA035: Bispecific antibody targeting 4-1BB and EGFR. 41BB is expressed on the surface of leucocytes and induces T cell proliferation, B cell antibody secretion and dendritic cell maturation upon its interaction with 41BBL.

Catumaxomab: trifunctional IgG2 antibody targeting CD3 and EpCAM. EpCAM is a surface antigen expressed on the cells of several tumor types. The modified Fc portion binds to FcR of APCs.

CBP-1008: Bispecific ligand/drug conjugate targeting folate-receptor alpha (FRα) and vanilloid subfamily member 6 of transient receptor potential channels (TRPV6) carrying monomethyl auristatin E (MMAE). Both FRα and TRPV6 are overexpressed in several solid tumors.

CC-1: Bispecific IgGsc antibody with prologed half-life targeting PSMA and CD3. PSMA is expressed in prostatic tissue and overexpressed in prostate cancer, as well as in other tumor types.

CD3-MUC1 Bispecific Antibody: Citokine induced killer (CIK) armed with a bispecific antibody targeting CD3 and MUC1. MUC1 is a transmembrane glycoprotein overexpressed in several carcinomas.

CDX-527: Tetravalent bispecific antibody formed by an IgG1 PD-L1 monoclonal antibody and the scFv of a CD27 agonist monoclonal antibody.

ABL001 / CTX-009 / NOV1501: Bispecific antibody that simultaneously target DLL4 and VEGF-A. DLL4 is associated with angiogenesis and stem cell homeostasis.

CX-904: Conditionally activated T-cell bispecific targeting EGFR and CD3.

EMB-01: Bispecific antibody targeting EGFR and c-MET.

EMB-02: Fabs-In-Tandem (FIT)-Ig bispecific antibody targeting PD-1 and LAG-3. LAG-3 is an inhibitory immune checkpoint expressed on activated T cells, NK cells, B cells and dendritic cells, which modulates T cell activities.

EMB-07: (Monovalent Assimetric Tandem) MAT-Fab bispecific antibody targeting CD3 and ROR1.

EMB-09: FIT-Ig tetravalent bispecific antibody targeting PD-L1 and OX40. OX40, when binding to OX40L, activates T cell and promotes expansion of effector and memory populations.

ERY974: Humanized IgG4 bispecific T cell-redirecting antibody (TRAB) targeting CD3 and GPC3. GPC3 is a heparan sulfate proteoglycan with high cancer specificity.

ES014: Bispecific antibody inhibiting CD39 and TGF-β. CD39 is a key regulator of the adenosine pathway and, along with TFG-β, are associated with immunosupression in the TME.

FS101: Tetravalent bispecific antibody targeting 4-1BB and PD-L1. 4-1BB is expressed on the surface of leucocytes and induces T cell proliferation, B cell antibody secretion and dendritic cell maturation upon its interaction with 4-1BBL.

FS120: Tetravalent bispecific antibody targeting 4-1BB and OX40. 4-1BB is expressed on the surface of leucocytes and induces T cell proliferation, B cell antibody secretion and dendritic cell maturation upon its interaction with 4-1BBL. OX40, when binding to OX40L, activates T cell and promotes expansion of effector and memory populations.

FS118: Tetravalent bispecific antibody targeting PD-L1 and LAG-3. LAG-3 is an inhibitory immune checkpoint expressed on activated T cells, NK cells, B cells and dendritic cells, which modulates T cell activities.

FS222: Tetravalent bispecific IgG1 antibody targeting PD-L1, with 4-1BB binding sites engineered into the Fc region. 4-1BB is expressed on the surface of leucocytes and induces T cell proliferation, B cell antibody secretion and dendritic cell maturation upon its interaction with 4-1BBL.

GEM3PSCA: Affinity-Tailored Adapter for T-Cells (ATAC) that simultaneously binds to CD3 and prostate stem cell antigen (PSCA). PSCA is expressed on the prostate, but also in other tissues as bladder, colon or stomach.

GEN1044: Bispecific IgG1 antibody targeting CD3 and 5T4. 5T4 is a oncofetal antigen present in several cancers but rarely expressed in healthy tissues.

GEN1046: Bispecific DuoBody® targeting PD-L1 and 4-1BB. 4-1BB is expressed on the surface of leucocytes and induces T cell proliferation, B cell antibody secretion and dendritic cell maturation upon its interaction with 4-1BBL.

GEN1047: Bispecific DuoBody® targeting CD3 and B7H4. B7H4 is an inhibitory immune checkpoint expressed on malignant cells.

HLX301: Recombinant humanized bispecific antibody against PD-L1 and TIGIT. TIGIT is an inhibitory receptor expressed in lymphocytes that interacts with CD155 on antigen-presenting cells, downregulating T cells and NK cells activities.

HLX35: Bispecific antibody targeting EGFR and 4-1BB. 4-1BB is expressed on the surface of leucocytes and induces T cell proliferation, B cell antibody secretion and dendritic cell

IBI318: Bispecific recombinant fully-human IgG1 antibody targeting PD-1 and PD-L1.

IBI322: Bispecific antibody targeting PD-L1 and CD47. CD47 inhibits the activity of macrophages through binding SIRPα.

IMA401: Bispecific T cell engaging receptor (TCER®) targeting Melanoma Associated Antigen 4/8 (MAGEA4/8) and CD3. MAGEA4/8 is overexpressed in different tumor types.

IMC-F106C: HLA-A*0201-restricted Immune-Mobilizing Monoclonal T Cell Receptor Against Cancer (ImmTAC ®) that simultaneously attaches to CD3 and PRAME. PRAME is expressed in several tumor types.

IMCnyeso: HLA-A*0201-restricted Immune-Mobilizing Monoclonal T Cell Receptor Against Cancer (ImmTAC ®) targeting NY-ESO-1/LAGE-A1 and CD3. NY-ESO-1/LAGE-A1 are cancer testis antigens expressed in several tumors types and with low expression in healthy tissue.

IMM2902: Recombinant bispecific antibody-Trap fusión protein targeting CD47 and HER2. CD47 inhibits the activity of macrophages through binding SIRPα.

INCA32459-101: Bispecific antibody targeting LAG-3 and PD-1.

INBRX-105: Recombinant humanized bispecific IgG antibody targeting PD-L1 and 4-1BB.

Ivonescimab: Bispecific antibody targeting PD-1 and VEGF

JNJ-78306358: T cell redirecting bispecific antibody (TRAB) targeting CD3 and HLA-G. HLA-G is a major histocompatibility class I molecule frequently expressed on the surface of cancer cells but with limited expression in healthy tissue.

KM257: Bispecific antibody targeting two different domains of HER2, it also induces antibody dependent cytotoxicity (ADCC).

KN026: Bispecific antibody generated from heavy chains of trastuzumab and pertuzumab, that simultaneously targets two different epitopes of HER2.

KN046: Recombinant humanized bispecific antibody targeting PD-L1 and CTLA-4.

LB1410: Recombinant humanized bispecific antibody targeting PD-1 and TIM-3. TIM-3 is an immunoregulatory protein expressed on T-cells, Tregs and other immune cells.

Lorigerlimab: Bispecific tetravalent IgG4 Dual-Affinity Re-Targeting antibody (DART®) that simultaneously inhibits PD-1 and CTLA-4.

LY3164530: Bispecific IgG4 antibody targeting MET with a single chain variable fragment (scFV) to EGFR fused to the N-terminus of each heavy chain.

LY3415244: Bispecific antibody targeting PD-L1 and TIM3. TIM-3 is an immunoregulatory protein expressed on T-cells, Tregs and other immune cells.

LY3434172: Bispecific human IgG1 antibody with a silent Fc region targeting PD-1 and PD-L1

M1231: Bispecific antibody-drug conjugate targeting MUC1 and EGFR. MUC1 is a transmembrane glycoprotein overexpressed in several carcinomas.

M701: Bispecific T-cell engager antibody targeting CD3 and EpCAM. EpCAM is a surface antigen expressed on the cells of several tumor types.

M802: Humanized bispecific antibody composed of a monovalent unit against HER2 and a scFV targeting CD3.

MBS301: Glyco-engineered bispecific IgG1 antibody generated from Trastuzumab and Pertuzumab targeting HER2.

MCLA-128 / Zenocutuzumab: ADCC enhanced humanized bispecific IgG1 antibody targeting HER2 and HER3, that blocks NRG1 binding and HER2/3 dimerization.

MCLA-129: Human bispecific antibody targeting EGFR and c-MET.

MCLA-145: Bispecific Fc-silenced IgG1 antibody targeting PD-L1 and 4-1BB. 4-1BB is expressed on the surface of leucocytes and induces T cell proliferation, B cell antibody secretion and dendritic cell maturation upon its interaction with 4-1BBL.

MCLA-158 / Petosemtamab: Bispecific IgG1 antibody targeting EGFR and LRG5. LRG5 is a receptor overexpressed in cancer stem cells.

MEDI5752: Monovalent bispecific antibody targeting PD-1 and CTLA-4, with preferential affinity for PD-1+ activated T cells.

Mitoxantrone packaged EDV (EnGeneIC Delivery Vehicle): Bacteria-derived nanocell loaded with Mitoxantrone and coated in bispecific antibodies targeting EGFR. After attachement to tumor cell, EDV is endocytosed and releases Mitoxantrone.

MM-111: Bispecific antibody fusion protein targeting HER2 and HER3.

MVC-101 / TAK-186: T cell engaging bispecific antibody and Conditional Bispecific Redirected Activation (COBRA) protein targeting EGFR and CD3. Contains a linker that is cleaved by proteases of the tumor microenvironment, unleashing the active protein, and a human serum albumin binding domain to extend the half-life.

NI-1801: Bispecific human IgG1 antibody targeting CD47 and MSLN. CD47 is a tumor-associated antigen usually found on the surface of healthy cells but overexpressed on cancer cells, which inhibits macrophage activation. MSLN is protein overexpressed on the surface of some carcinomas including mesothelioma and ovarian cancer.

Nivatrotamab: Bispecific antibody comprised of a humanized anti-CD3 scFv attached to a humanized GD2 targeted IgG1 light chain. GD2 is a disialoganglioside expressed in several tumors including neuroblastoma and melanoma, with low expression in healthy tissues.

PF-07257876: Bispecific antibody targeting PD-L1 and CD47.

PM1009: Bispecific human IgG1 monoclonal antibody targeting T cell immunoglobulin and ITIM domain (TIGIT), fused with a scFv against poliovirus receptor-related immunoglobulin (PVRIG). TIGIT is an inhibitory receptor expressed in lymphocytes that interacts with CD155 on antigen-presenting cells, downregulating T cells and NK cells activities. PVRIG inhibits T cell receptor signaling upon interaction with NECTIN2.

PM8001: Protein composed of the extracellular domain of the TGF-β RII, that causes TGF-β blockade, fused to a humanized IgG1 antibody against PD-L1. TGF-β is associated with immunosuppression in the tumor microenvironment.

PRS-343: Bispecific antibody-Anticalin® fusion targeting HER-2 and 4-1BB. Anticalin are recombinant human proteins based on lipocalins. 4-1BB is expressed on the surface of leucocytes and induces T cell proliferation, B cell antibody secretion and dendritic cell maturation upon its interaction with 4-1BBL.

PRS-344/S095012: Bispecific antibody-Anticalin® fusion targeting PD-L1 and 4-1BB. Anticalin are recombinant human proteins based on lipocalins. 4-1BB is expressed on the surface of leucocytes and induces T cell proliferation, B cell antibody secretion and dendritic cell maturation upon its interaction with 4-1BBL.

PT217: Bispecific antibody targeting DLL3 and CD47. DLL3 is an inhibitory Notch ligand overexpressed in neuroendocrine tumors. CD47 is a tumor-associated antigen usually found on the surface of healthy cells but overexpressed on cancer cells, which inhibits macrophage activation.

OMP-305B83 / Navicixizumab: Bispecific humanized IgG2 antibody targeting DLL4 and VEGF. DLL4 is associated with angiogenesis and stem cell homeostasis.

Q-1802: Bispecific humanized antibody targeting Claudin 18.2 and PD-L1. Claudin 18.2 is a member of the claudin family frequently expressed in different cancers and with low expression on healthy tissue.

QLS31901: Bispecific protein composed of the extracellular domain of the TGF-β RII, that causes TGF-β blockade, fused to a humanized IgG1 antibody against PD-L1. TGF-β is associated with immunosuppression in the tumor microenvironment.

QLS31904: Recombinant protein composed of two humanized binding domains from antibodies targeting DLL3 and CD3. DLL3 is an inhibitory Notch ligand overexpressed in neuroendocrine tumors.

REGN7075: Bispecific human IgG4 antibody targeting EGFR and CD28, favouring physical contact of activated CD28 positive T cells and EGFR expressing tumor cells. CD28 is a costimulatory molecule required for an effective immune response.

RO6958688: Bispecific antibody targeting carcinoembryonic antigen (CEA) and CD3. CEA is a surface glycoprotein frequently overexpressed in gastrointestinal neoplasms.

RO7121661 / Tobemstomig: Bispecific antibody targeting PD-1 and TIM-3. TIM-3 is an immunoregulatory protein expressed on T-cells, Tregs and other immune cells.

RO7247669 / Lomvastomig: Bispecific IgG1-based antibody with a silent Fc region targeting PD-1 and LAG-3. LAG-3 is an inhibitory immune checkpoint expressed on activated T cells, NK cells, B cells and dendritic cells, which modulates T cell activities.

SI-B001: Specificity Enhanced Bispecific Antibody (SEBA) tetravalent antibody targeting EGFR and HER3, with an active Fc receptor able to mediate innate responses against cancer cells.

SI-B003: Specificity Enhanced Bispecific Antibody (SEBA) tetravalent antibody targeting PD-L1 and CTLA-4.

TargomiRs: Minicells containing a miR-16-based microRNA, a drug delivery vehicle (EDV) and a bispecific EGFR targeted antibody. miR-16 has been linked to tumor suppression.

Tarlatamab: Bispecific T cell engager targeting CD3 and DLL3. DLL3 is an inhibitory Notch ligand overexpressed in neuroendocrine tumors.

Tebotelimab: Bispecific tetravalent Fc-bearing Dual Affinity Re-Targeting (DART®) humanized antibody targeting PD-L1 and LAG-3. LAG-3 is an inhibitory immune checkpoint expressed on activated T cells, NK cells, B cells and dendritic cells, which modulates T cell activities.

TF2: Tri-Fab bispecific antibody targeting carcinoembryonic antigen (CEA) and histamine-succinyl-glycine (HSG) peptide hapten. CEA is a surface glycoprotein frequently overexpressed in gastrointestinal neoplasms. A radionuclide attached HSG peptide-hapten is administered, allowing CEA-expressing cells detection.

TJ033721: Bispecific antibody targeting Claudin 18.2 and 4-1BB. Claudin 18.2 is a member of the claudin family frequently expressed in different cancers and with low expression on healthy tissue. 4-1BB is expressed on the surface of leucocytes and induces T cell proliferation, B cell antibody secretion and dendritic cell maturation upon its interaction with 4-1BBL.

TQB2930: Bispecific antibody targeting two different epitopes of HER2.

TST005: Protein composed of the extracellular domain of the TGF-β RII, that causes TGF-β blockade, fused to a humanized IgG1 antibody against PD-L1. TGF-β is associated with immunosuppression in the tumor microenvironment.

XmAb18087 / Tidutamab: Bispecific humanized antibody targeting CD3 and SSTR2, with a full Fc domain. SSTR2 is expressed in many tumors, particularly in neuroendocrine neoplasms.

XmAb20717 / Vudalimab: Bispecific humanized antibody targeting PD-1 and CTLA-4, with engineered Fc domain to avoid FcγR interactions.

XmAb22841: Bispecific humanized antibody targeting LAG-3 and CTLA-4, with engineered Fc domain to increase stability and half life. LAG-3 is an inhibitory immune checkpoint expressed on activated T cells, NK cells, B cells and dendritic cells, which modulates T cell activities.

XmAb23104 / Izuralimab: Bispecific antibody targeting T cells that express both PD-1 and Inducible T-Cell Costimulator (ICOS). ICOS is a costimulatory protein of the CD28 family, with a main role on the immune response.

XmAb808: Bispecific antibody targeting B7-H3 and CD28. CD28 is a costimulatory molecule required for an effective immune response. B7-H3 is a tumor antigen widely expressed on the surface of tumor cells.

Y101D: Recombinant bispecific antibody targeting PD-L1 and TGF-β. TGF-β is associated with immunosuppression in the tumor microenvironment.

YH32367: Bispecific humanized IgG1 antibody targeting HER2 and 4-1BB. 4-1BB is expressed on the surface of leucocytes and induces T cell proliferation, B cell antibody secretion and dendritic cell maturation upon its interaction with 4-1BBL.

Zanidatamab: Bispecific humanized IgG1 antibody targeting two distinct domains of HER2.

ZW49: Bispecific antibody-drug conjugate attached to auristatin toxin with a protease-cleavable linker, targeting two distinct HER2 epitopes.
